# Fibrinogen: A Feasible Biomarker in Identifying the Severity and Acute Exacerbation of Chronic Obstructive Pulmonary Disease

**DOI:** 10.7759/cureus.16864

**Published:** 2021-08-03

**Authors:** Mikash Mohan, Ashwaghosha Parthasarathi, Chaya S K, Jayaraj Biligere Siddaiah, Padukudru A Mahesh

**Affiliations:** 1 Department of Pulmonology, Jagadguru Sri Shivarathreeshwara Medical College, Mysore, IND; 2 Department of Epidemiology and Public Health, Allergy Asthma and Chest Center, Mysore, IND

**Keywords:** fibrinogen, inflammation, copd, aecopd, biomarker

## Abstract

Background

Chronic obstructive pulmonary disease (COPD) is no longer considered a disease exclusive to the respiratory system. It is a multipronged disease with both lung and systemic involvement. Although the forced expiratory volume (FEV) in one second is one of the most commonly used markers to assess disease severity, in recent years, biomarkers such as interleukin-1 beta, serum C-X-C motif chemokine ligand 10, ﬁbrinogen, soluble receptor for advanced glycation, surfactant protein D, and club cell secretory protein have been proven to be effective markers to assess disease severity.

Objective

The current study aimed to test the association of fibrinogen levels with increased exacerbation of COPD per year and lower lung function and to discuss its potential utility as a biomarker.

Methodology

A total of 105 participants were enrolled in the study. The study participants included 35 stable COPD patients, 35 COPD patients with acute exacerbation, and 35 non-COPD healthy controls (matched for age and gender). All patients above 18 years of age who were diagnosed with COPD as per the Global Initiative for Chronic Obstructive Disease (GOLD) guidelines were considered for inclusion in the study. The patients were divided into stable COPD group and acute exacerbations of COPD (AECOPD) group based on the Anthonisen criteria. Sociodemographic factors, six-minute walk test, Medical Research Council Dyspnea Scale, and COPD Assessment Test scale were computed. Spirometry according to the American Thoracic Society guidelines and hematological investigations including serum fibrinogen were performed. Additionally, GOLD staging and severity indices were used to determine the clinical phenotyping of COPD, namely, ADO (age, dyspnea, airflow obstruction) index, BODE (body mass index, airflow obstruction, dyspnea, and exercise capacity) index, and DOSE (dyspnea, obstruction, smoking, exacerbation) index.

Results

Plasma fibrinogen level was significantly higher in the COPD groups compared to the control group. Plasma fibrinogen level was elevated in AECOPD compared to stable COPD patients. In addition, fibrinogen levels showed a positive correlation with important functional indices and prognostic markers such as BODE, ADO, and DOSE indices and a negative correlation with lung function. The odds of predicting an acute exacerbation of COPD for patients with FEV of <50% and FEV of >50% were 17.2 (area under the curve [AUC] = 0.825; sensitivity = 90.4%; specificity = 62.79%) and 15.1 (AUC = 0.791; sensitivity = 57.7%; specificity = 92.5%), respectively.

Conclusions

Plasma fibrinogen has the potential to be an important biomarker in the management of COPD and its exacerbation due to its ability to be responsive to the COPD disease statuses such as the severity of COPD and AECOPD.

## Introduction

Chronic respiratory diseases such as chronic obstructive pulmonary disease (COPD) are among the leading causes of morbidity and mortality worldwide [1,2]. Globally, it is estimated that 5% of all deaths are attributed to COPD, of which more than 90% occur in low-­ and middle-income countries (LMICs) [2]. COPD is no longer considered a disease exclusive to the respiratory system. It is a multipronged disease with both lung and systemic involvement [3].

Forced expiratory volume in one second (FEV1) is the marker most commonly associated with COPD. However, FEV1 is known to correlate inadequately with symptoms of COPD. This is mainly because the FEV1 values are based on the severity of airflow obstruction which is known to inaccurately predict the occurrence of exacerbations [4].

COPD, especially in the latter stages of the disease, is frequently associated with exacerbations. Acute exacerbation of COPD (AECOPD) is defined as “an acute worsening of respiratory symptoms requiring a change in treatment” [5]. These exacerbations are a known contributor to diminished lung function, quality of life, and mortality rates [6-8], and the prevention of COPD exacerbations is a vital therapeutic goal. Recent systematic reviews on the association of protein biomarkers with COPD outcomes identified the emergence of interleukin-1 beta, serum C-X-C motif chemokine ligand 10, ﬁbrinogen, soluble receptor for advanced glycation, surfactant protein D, and club cell secretory protein as novel biomarkers [9]. Plasma fibrinogen levels are of great importance, especially in LMICs, as they are economically feasible, easy to measure, and repeated as needed in routine clinical practice [10]. The goal of this study was to evaluate the association of serum fibrinogen levels with COPD outcomes and the effectiveness of serum fibrinogen levels and their optimal cut-off values as prognostic biomarkers for hospitalized COPD patients.

The current study aimed to test the hypothesis that elevated fibrinogen levels are associated with increased AECOPD cases per year, lower lung function, and lower functional indices such as BODE (body mass index, airflow obstruction, dyspnea, and exercise capacity), ADO (age, dyspnea, airflow obstruction), and DOSE (dyspnea, obstruction, smoking, exacerbation). In addition, it tests the effectiveness of serum fibrinogen levels and their optimal cut-off values to predict episodes of acute exacerbation for hospitalized COPD patients with moderate and severe COPD.

## Materials and methods

Study population

Participants were recruited from a tertiary care university teaching hospital from November 2019 to April 2020. The Institutional Ethics Committee of JSS Medical College, Mysore approved this study. This study included 35 stable COPD patients, 35 COPD patients with acute exacerbation, and 35 non-COPD healthy controls, who were matched for both age and gender.

All patients above 18 years of age who were diagnosed with COPD as per the Global Initiative for Chronic Obstructive Disease (GOLD) guidelines were considered for inclusion in the study. The patients were divided into stable COPD group and AECOPD group based on the Anthonisen criteria, which comprise three patient-reported items, namely, increased dyspnea, increased sputum volume, and increased sputum purulence [11].

Patients were excluded from the study if they failed to give consent; had any other respiratory disease other than COPD; were diagnosed with disseminated intravascular coagulopathy, pulmonary embolism, or deep vein thrombosis; or were on regular oral steroids and anticoagulants. Additionally, for the stable COPD group, patients who were currently stable but had an acute exacerbation within the previous month were excluded.

Laboratory and clinical assessments

Sociodemographic factors such as age, gender, body mass index (BMI), history of asthma exacerbation, and history of smoking were gathered from patient files. The six-minute walk test was performed on the day of the outpatient department (OPD) visit for those with stable COPD, while the AECOPD patients were tested on the day of discharge. Medical Research Council Dyspnea Scale (mMRC) and COPD Assessment Test (CAT) scale were used to assess the respiratory symptoms on the day of admission or OPD visit recorded by the resident physician [12,13].

On the day of admission or the OPD visit, 2 mL of venous blood sample was drawn and data from a hematological panel, conducted using an automated blood analyzer Sysmex XN 1000 (Sysmex Corp., Kobe, Japan), were recorded. In addition, absolute neutrophile count/absolute lymphocyte count and PLR platelet count/absolute lymphocyte count were calculated. Serum fibrinogen levels were assessed by turbidimetric immunoassay using KinesisDx kit (KinesisDx, Brea, CA) (assay range: 0.6-9.6 mg/mL) according to the standard protocols. Spirometry was performed on the same day using EasyOne® spirometer (NDD Medical Technologies, Zurich, Switzerland) according to American Thoracic Society guidelines [14].

Comorbidities in the patient group were recorded using the Charlson comorbidity index (CCI) and age-adjusted CCI [15]. Additionally, GOLD staging and severity indices were used to determine the clinical phenotyping of COPD, namely, ADO index, BODE index, and DOSE index [16].

Statistical analysis

Statistical analysis was done using Jamovi (v1.6, The jamovi project). Continuous variables were presented as either mean ± standard deviation (SD). Categorical variables were presented as percentages. Statistical significance between categorical variables was assessed using the chi-square test and by Student’s t-test for continuous variables. Correlation between plasma fibrinogen levels and functional indices such as BODE, ADO, and DOSE was calculated using Pearson’s correlation coefficient. Patients were further stratified into those with fibrinogen levels of <350 mg/dL and >350 mg/dL. This threshold was based on an integrated database at the participant level from five individual studies conducted by the COPD Biomarkers Qualification Consortium [17].

The area under the curve (AUC), sensitivity, specificity, odds ratio (OR), and optimal cut-off values were calculated based on the receiver operating characteristic (ROC) curve for groups with moderate (FEV1 of >50%) and severe (FEV1 of <50%) COPD. A p-value of <0.05 was considered statistically significant.

## Results

A total of 105 patients were enrolled in the study. The patients were divided into three groups: healthy controls, stable COPD patients, and AECOPD patients, with 35 participants in each group. The mean age of controls was 59.5 ± 13.1, while the mean ages of stable COPD and AECOPD patients were 60.7 ± 9.87 and 63.7 ± 9.28 years. The study sample consisted predominantly of males (n = 101; 95.2%). Overall, 85.8% (n = 91) participants had a smoking history. The age-adjusted CCI score was 7.23 ± 1.75 and 3.37 ± 3.75 in the stable COPD group and 3.33 ± 1.63 in the control group.

**Figure 1:**
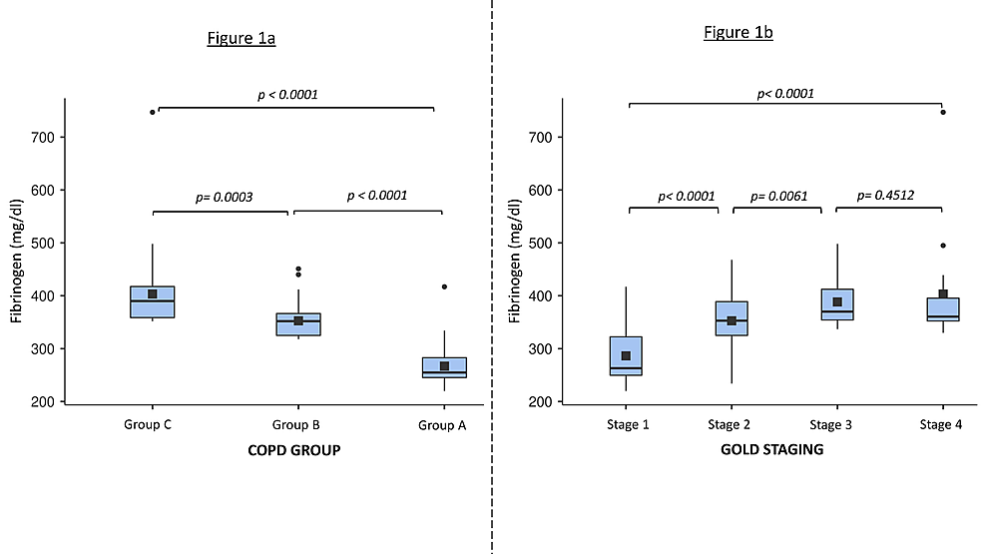
Plasma fibrinogen levels in the patient population. a: Box plot illustrating plasma fibrinogen levels between different COPD groups. The lower and upper borders of the box plot illustrate the Q1 and Q3 quartile. The solid square in the box illustrates the mean, for which the test of significance was calculated. Data outside the Q1–Q3 range are plotted as dots (outliers of the data). Group A: healthy individuals; Group B: individuals with stable COPD; Group C: individuals with acute exacerbation of COPD. b: Box plot illustrating plasma fibrinogen levels between different COPD groups differentiated by GOLD staging. The lower and upper borders of the box plot illustrate the Q1 and Q3 quartiles. The solid square in the box illustrates the mean, for which the test of significance was calculated. Data outside the Q1–Q3 range are plotted as dots (outliers of the data). Stage 1: FEV1, ≥80%; Stage 2: FEV1, 50-79%; Stage 3: FEV1, 30-49%; Stage 4: FEV1: ≤30%. COPD: chronic obstructive pulmonary disease; GOLD: Global Initiative for Chronic Obstructive Disease; FEV1: forced expiratory volume in one second

Lung function was more impaired in the AECOPD than the stable COPD group, as assessed using percentage predicted FEV1 (stable COPD = 62.3 ± 17.1; AECOPD = 50.6 ± 15.8; p = 0.004). Total leukocyte count, neutrophils, and fibrinogen levels were higher in the AECOPD group (p < 0.001). Further details on the study population are listed in Table 1. Plasma fibrinogen level in the control group was the lowest at 267 ± 37.2 mg/dL followed by 353 ± 32.2 mg/dL in stable COPD and 405 ± 71.6 mg/dL in AECOPD groups (Figure 1a). Similarly, fibrinogen levels were the lowest in GOLD stage 1 patients (286 ± 51.7 mg/dL) and the highest in stage 4 (403 ± 108 mg/dL) (Figure 1b).

**Table 1:** Baseline characteristics of the study participants. COPD: chronic obstructive pulmonary disease; AECOPD: acute exacerbation of chronic obstructive pulmonary disease; BMI: body mass index; FVC: forced vital capacity; FEV1: forced expiratory volume in one second; 6MWD: six-minute walk distance; TLC: total leucocyte count

COPD group	Controls (n = 35)	Stable COPD (n = 35)	AECOPD (n = 35)
	Mean ± SD	Mean ± SD	Mean ± SD
Age	59.5 ± 13.1	60.7 ± 9.94	63.8 ± 9.4
Gender (M/F)	33/2	34/1	32/3
BMI	25.5 ± 3.87	21.5 ± 4.25	22.4 ± 3.44
Smoking history			
Nonsmoker	35 (100%)	3 (8.57%)	1 (2.85%)
Current smoker	0	32 (91.42%)	34 (97.14%)
Charlson comorbidity index			
Unadjusted	2.01 ± 1.04	2.03 ± 2.06	3.97 ± 1.07
Age-adjusted	3.33 ± 1.63	3.37 ± 3.75	7.23 ± 1.75
Pulmonary function tests			
6MWD (m)	N/A	439 ± 36.5	330 ± 88.3
FEV1 (L)	2.28 ± 0.58	1.34 ± 0.465	1.15 ± 0.37
FEV1 (%)	91 ± 13.3	60.7 ± 17	51.4 ± 14.6
FEV1 (% predicted)	90.3 ± 14.1	62.3 ± 17.1	50.6 ± 15.8
FEV1/FVC (%)	97.3 ± 4.94	73.7 ± 9.57	68.0 ± 11.8
FEV1/FVC (% predicted)	98.1 ± 7.91	76 ± 9.34	67.9 ± 10.3
Hematological tests			
Hemoglobin (g/dL)	13.7 ± 2.33	14.3 ± 2.31	14.5 ± 1.93
Packed cell volume (%)	39.8 ± 5.58	42.8 ± 8.49	45.5 ± 6.7
TLC (cells/mm^3^)	7,998 ± 2,509	9,306 ± 2,380	12,155 ± 5,544
Neutrophils (cells/mm^3^)	5,053 ± 2,219	7,209 ± 2,873	10,287 ± 6,089
Lymphocytes (cells/mm^3^)	2,237 ± 735	1,413 ± 856	1,334 ± 784
Platelets (10^6^/mm^3^)	2.7 ± 0.817	2.47 ± 0.67	2.51 ± 0.855
Fibrinogen (mg/dL)	267 ± 37.2	353 ± 32.2	405 ± 71.6
Eosinophils (cells/mm^3^)	NA	201 ± 209	256 ± 492

Binomial multivariable analysis of various risk factors between both the control and the COPD group and the stable COPD and AECOPD group showed fibrinogen as an independent risk factor in both scenarios (adjusted odds ratio: 2.9 [2.27, 3.71]; 1.7 [1.37, 2.12]) (Table 2).

**Table 2:** Binomial multivariable analysis for independent risk for different models. Model 1: binomial multivariable analysis was done between healthy controls and COPD patients. Model 2: binomial multivariable analysis was done between stable COPD patients and AECOPD patients. * indicates a statistically significant outcome with a p-value < 0.05. AOR: adjusted odds ratio; BMI: body mass index; CCI: Charlson comorbidity index; 6MWD: six-minute walk distance; FVC: forced vital capacity; FEV1: forced expiratory volume in one second; TLC: total leukocyte count

COPD group	Model 1	Model 2
	AOR (95% CI)	AOR (95% CI)
Age	1.04 (0.85-1.28)	1.35 (0.88-2.7)
Gender	1.48 (0.98-2.10)	1.43 (0.95-2.03)
BMI	1.08 (0.68-1.71)	1.10 (0.70-1.74)
Smoking (ref: Y)	4.39 (4.00-4.82)*	5.10 (3.81, 6.81)*
Age-adjusted CCI	1.02 (0.84, 1.24)	2.09 (0.9, 5.21)
Pulmonary function tests		
6MWD (m)	NA	1.2 (0.41, 3.47)
FEV1 (L)	2.32 (1.2, 4.5)*	1.94 (1.03, 3.67)*
FEV1/FVC	3.92 (3.58-4.30)*	3.97 (3.62-4.36)*
Hematological tests		
Hemoglobin (g/dL)	1.39 (0.71, 2.71)	1.65 (0.86, 3.15)
Packed cell volume (%)	1.03 (0.56, 1.87)	1.12 (0.61, 2.04)
TLC (cells/mm^3^)	2.19 (1.22-3.91)*	1.44 (0.95, 2.19)
Neutrophils (cells/mm^3^)	1.4 (0.51, 3.88)	2.2 (0.88, 5.5)
Lymphocytes (cells/mm^3^)	1.01 (0.79, 1.28)	2.18 (0.9, 5.29)
Platelets (10^6^/mm^3^)	1.58 (0.82, 3.02)	1.2 (0.74, 1.97)
Fibrinogen (mg/dL)	2.9 (2.27, 3.71)*	1.7 (1.37, 2.12)*
Eosinophils (cells/mm^3^)	NA	1.32 (0.73, 2.37)

Patient stratification based on fibrinogen levels

The patients were divided into two groups based on fibrinogen levels: fibrinogen <350 mg/dL (n = 51) and fibrinogen ≥350 mg/dL (n = 54). No significant difference was observed in age-adjusted CCI (p = 0.939) between the two groups. The high fibrinogen group had a higher mMRC dyspnea grade (p < 0.001) and CAT score than the low fibrinogen group (p = <0.001). The high fibrinogen group included patients with a greater number of yearly acute exacerbations (p < 0.001). GOLD stage D was the most common in both groups (n = 33.9%). The two groups showed significant differences in the GOLD COPD indices (p = 0.029). The decline in lung function, comprising FEV1 (p = 0.017), FVC (p = 0.007), and distance of the six-minute walk test (p = 0.016), was considerably worse in the high fibrinogen group than in the low fibrinogen group. Further details are listed in Table 3.

**Table 3:** Pulmonary findings of patients stratified using plasma fibrinogen levels. GOLD staging: Stage 1: FEV1, ≥80%; Stage 2: FEV1, 50-79%; Stage 3: FEV1, 30-49%; Stage 4: FEV1, ≤30%. CCI: Charlson comorbidity index; mMRC: modified Medical Research Council Dyspnea; CAT: COPD Assessment Test; AE: acute exacerbations; FVC: forced vital capacity; FEV1: forced expiratory volume in one second; 6MWD: Six-minute walk distance

	Fibrinogen <350 mg/dL (n = 51)	Fibrinogen ≥350 mg/dL (n = 54)	
	Mean ± SD	Mean ± SD	P-value
Age-adjusted CCI	3.63 ± 3.68	3.58 ± 2.88	0.939
mMRC scoring	2.13 ± 0.51	2.98 ± 0.98	<0.001
GOLD staging			
Stage 1 (%)	18 ± 35.90	10 ± 17.90	0.029
Stage 2 (%)	17 ± 33.30	12 ± 22.40	
Stage 3 (%)	1 ± 2.60	2 ± 4.50	
Stage 4 (%)	14 ± 28.20	20 ± 37.30	
CAT score	16.9 ± 9.1	23.7 ± 10.4	<0.001
Number of AE per year	1.6 ± 0.69	1.9 ± 0.58	<0.001
BODE index	3.4 ± 1.45	4.11 ± 1.24	0.009
ADO index	3.2 ± 1.42	4.95 ± 1.4	<0.001
DOSE index	2.9 ± 0.87	4.08 ± 1.45	<0.001
FVC (L)	2.14 ± 0.68	2.11 ± 0.44	0.007
FEV1 (L)	1.35 ± 0.51	1.23 ± 0.38	0.017
FEV1/FVC	0.62 ± 0.08	0.58 ± 0.09	0.029
6MWD (m)	346 ± 39.6	329 ± 67.2	0.016

Correlation among chronic obstructive pulmonary disease severity and plasma fibrinogen

**Figure 2:**
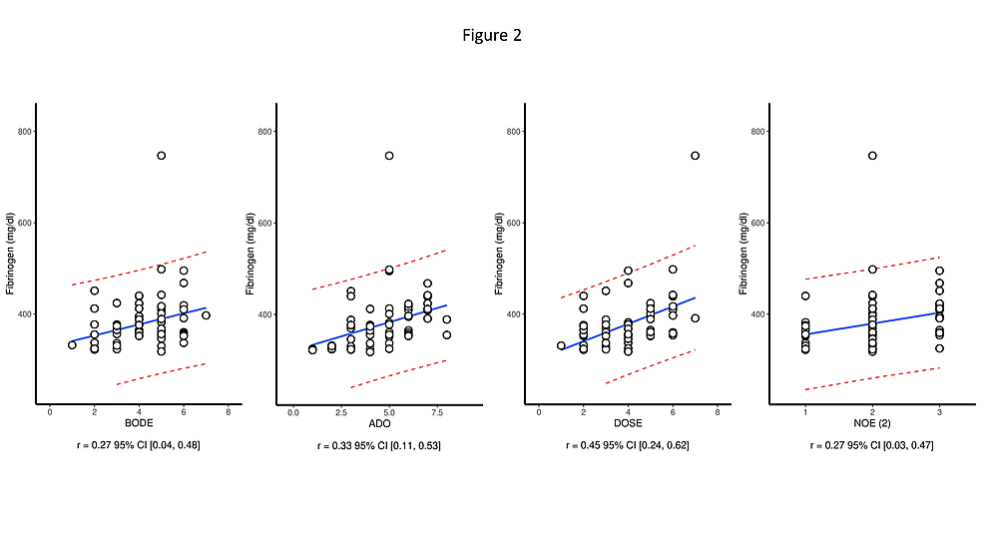
Correlation between the COPD severity index and the level of fibrinogen. COPD: chronic obstructive pulmonary disease; DOSE: dyspnea, obstruction, smoking, exacerbation; BODE: body mass index, airflow obstruction, dyspnea, exercise; ADO: age, dyspnea, airflow obstruction; NOE: number of exacerbations per year

In addition, we found a significant positive correlation between fibrinogen level and the number of exacerbations per year (r = 0.27, p = 0.005) and a negative correlation with FEV1 (r = 0.613, p < 0.001). The fibrinogen level revealed statistically significant positive correlations with BODE index (r = 0.27, p = 0.05), ADO index (r = 0.33, p < 0.001), and DOSE index (r = 0.45, p < 0.001) (Figure 2).

Prognostic accuracy of fibrinogen

The mean fibrinogen level in stable COPD patients was lower than AECOPD (353 ± 31.9 mg/dL vs 405 ± 71.1 mg/dL; p < 0.01). The overall cut-off for fibrinogen was 358 mg/dL with a sensitivity of 80% and specificity of 68.57% for all COPD patients (AUC = 0.820; odds of predicting AECOPD = 19.6). Prognostic accuracy was further calculated after being stratified into COPD patients as group A (FEV1 of <50%) with 26 patients and group B (FEV1 of >50%) with 44 patients.

**Figure 3:**
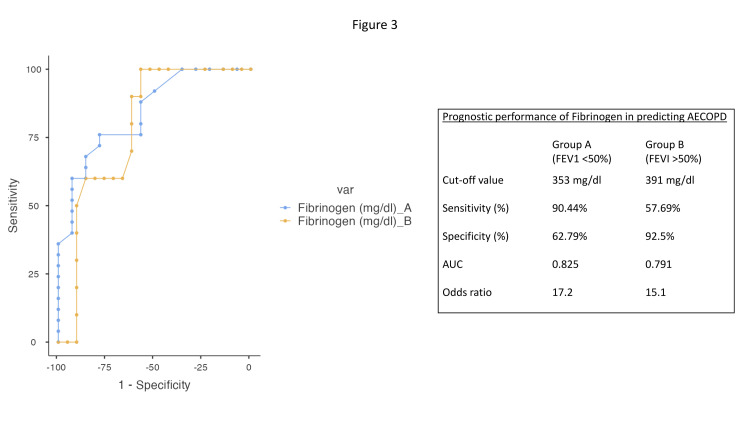
ROC analysis for plasma fibrinogen levels for Group A and B. Group A: FEV1 <50%; Group B: FEV1 >50%. AECOPD: acute exacerbation of chronic obstructive pulmonary disease; FEV1: forced expiratory volume in one second; AUC: area under the curve; ROC: receiver operating characteristic

In group A, a fibrinogen cut-off of 353 mg/dL had a sensitivity of 90.4% and specificity of 62.79% (using Youden’s index), an AUC of 0.825, and the odds of 17.2 for predicting acute COPD exacerbations. In group B, the cut-off was higher at 391 mg/dL with an AUC of 0.791 (sensitivity: 57.7%; specificity: 92.5%), and the odds of predicting acute COPD exacerbations was 15.1 (Figure 3).

## Discussion

We observed that plasma fibrinogen levels were the highest in the AECOPD group and significantly different from those with stable COPD or healthy controls. In addition, plasma fibrinogen levels had a positive correlation with important functional indices and prognostic markers such as the BODE, ADO, and DOSE. The best correlation was with the DOSE index and the least was with the BODE index. Furthermore, elevated fibrinogen levels were associated with an increased number of AECOPD per year and were significantly associated with lower lung function. Significant differences were observed in fibrinogen levels between mild and moderate COPD and moderate and severe COPD, but not between severe and very severe COPD. Fibrinogen levels of >350 mg/dL had a 90% sensitivity but lower specificity to predict AECOPD in severe COPD patients (FEV1 of <50%), while fibrinogen levels of >390 mg/dL had more than 90% specificity to predict AECOPD but had poor sensitivity in moderate COPD patients (FEV1 of >50%).

Fibrinogen, a major acute-phase reactant, is predominantly synthesized in the liver. Fibrinogen is then converted into fibrin by thrombin for blood coagulation [18]. The synthesis of fibrinogen is upregulated as a response to inflammation which is a key pathophysiology underlying COPD [19]. COPD, a systemic disease, comprises various phenotypes making disease progression and outcome difficult to predict [20-22]. Multiple cohorts including Evaluation of COPD Longitudinally to Identify Predictive Surrogate Endpoints (ECLIPSE), National Health and Nutrition Examination Survey III (NHANES III) study, and Framingham Heart Study Offspring Cohort (FHSOC) concluded that plasma fibrinogen levels can be used as a prognostic biomarker to assess AECOPD [16]. The U.S. Food and Drug Administration and the European Medicines Agency have approved plasma ﬁbrinogen as a prognostic biomarker to assess AECOPD and mortality in COPD confirming its clinical utility [23]. However, it should be noted that the demographics from these large cohorts consisted mainly of the Caucasian population. Because plasma fibrinogen levels are known to vary with demographics and ethnicities, the results from other cohorts may have varied applicability [24]. The mean fibrinogen levels from our study were similar to the NHANES III cohort study but lower than the ECLIPSE study and higher than Atherosclerosis Risk in Communities and Cardiovascular Health Study studies. This can be attributed to the differences in assessment methods for plasma fibrinogen levels or differences in the severity of COPD and exacerbators in the study population or differences in ethnicity [25]. For plasma fibrinogen levels to be accepted as a biomarker, it is crucial to standardize its measurement according to ethnicity and disease severity.

Similar to our study, large-scale longitudinal cohort studies performed in the general population observed that patients with higher plasma fibrinogen levels had increased rates of COPD exacerbations and hospitalization rates during the follow-up period [26,27]. We also saw a negative correlation between FEV1 and plasma fibrinogen levels and a positive correlation with various COPD-related indices (BODE, ADO, and DOSE). These findings were congruent with a similar study performed among COPD patients from South Korea [28]. The correlation between plasma fibrinogen and the various prognostic indices were only mild to moderate suggesting that fibrinogen may measure different aspects of the disease. Future studies are needed to confirm whether the addition of fibrinogen to the indices (for example, BODEF) is an even better indicator of disease outcomes.

In addition, plasma fibrinogen levels may be useful in decision-making concerning the consideration of antimicrobial agents in the treatment of AECOPD. Studies observed that a rise in plasma fibrinogen levels was more marked in the presence of symptoms consistent with an infective etiology, especially those of viral etiology [29]. Steroids are an important part of the management of AECOPD, and treatment with steroids has shown a significant decrease in plasma fibrinogen than in patients in nonsteroid therapy [30].

A novel finding of our study is the calculation of predictive accuracy of plasma fibrinogen levels in AECOPD in moderate and severe COPD patients. We found that in both severe (AUC: 0.82; OR: 17.2) and moderate (AUC: 0.79; OR: 15.1) COPD, elevated plasma fibrinogen was found to be significantly associated with risk of exacerbation. Larger and longitudinal studies assessing the use of plasma fibrinogen as a predictive tool for patients with different severities of COPD are required.

Overall, we observed that plasma fibrinogen is independently associated with COPD and its severity, exacerbations, and frequent exacerbators. Though we have not been able to test the responsiveness of the biomarker during stable disease and exacerbations in the same patient, previous studies have demonstrated in longitudinal studies that plasma fibrinogen is responsive to disease stability [22]. This would help place this biomarker in a position to fill an important lacuna.

The GOLD guidelines have a clear strategy on when to escalate the treatment but are not clear on when to de-escalate. Early de-escalation could lead to relapse of symptoms and another exacerbation. There is a need to identify a biomarker that could guide de-escalation. Future longitudinal studies are needed to critically evaluate whether serial plasma fibrinogen levels can be used to inform de-escalation in AECOPD patients. 

The main strengths of the study include a well-characterized study population. Diagnosis of COPD and its severity were determined according to the GOLD guidelines. To our knowledge, there is no study conducted in the Indian population investigating the association and predictive power of serum fibrinogen in COPD, its severity, and its exacerbation adding to the scientific literature information from a different ethnic population. However, there are a few notable limitations to the study. Our data were from a single center, including relatively small sample size. The distribution of gender in the study was greatly skewed toward males and was reflective of tobacco smoking COPD which may not be representative of the COPD population in India. The subjects were drawn from the OPD and patients admitted to a tertiary care center and may not be reflective of the COPD patients in the community.

## Conclusions

Plasma fibrinogen has the potential to be an important biomarker in the management of COPD and its exacerbations due to its ability to be responsive to COPD disease statuses such as the severity of COPD and AECOPD. Longitudinal studies in real-world clinical situations are needed to assess and understand the relevance of plasma fibrinogen as a biomarker for COPD, disease severity, and exacerbations of COPD, and whether it can guide de-escalation of treatment in AECOPD.
